# Letter to the editor: HIV in women in the World Health Organization (WHO) European Region

**DOI:** 10.2807/1560-7917.ES.2020.25.4.2000061

**Published:** 2020-01-30

**Authors:** Karoline Aebi-Popp, Fiona Mulcahy, Yvonne Gilleece, on behalf of WAVE

**Affiliations:** 1 Department of Infectious Diseases, University Hospital Bern, Switzerland; 2 Department of Infectious Diseases, St. James`s Hospital Dublin, Ireland; 3 Department of HIV Medicine, Brighton & Sussex University Hospitals NHS Trust, United Kingdom; 4 Women Against Viruses in Europe: https://www.eacsociety.org/wave/about-wave/wave.html

**Keywords:** HIV, prevention, PrEP in women

**To the editor**: We read with interest the article *HIV among women in the WHO European Region – epidemiological trends and predictors of late diagnosis, 2009-2018* by Mårdh et al., published on 28 November 2019 in *Eurosurveillance*, in which they report that in 2018, nearly 50,000 women were newly diagnosed with HIV, making up one-third of the new diagnoses, and new diagnoses occurred mainly in the East of the World Health Organization (WHO) European Region. Authors further noted late diagnoses in over half of cases and heterosexual transmission as the predominant route of infection [1].

We would like to point out that late diagnosis of HIV in the Region is largely driven by low coverage and uptake of HIV testing. A study from the United Kingdom showed that opportunities for HIV testing among women who have sexual risk are being missed in existing health facilities [2]. HIV testing based on HIV indicator conditions, such as other sexually transmitted infections, viral hepatitis, tuberculosis or certain cancers, is still not identifying the majority of new HIV diagnoses. Furthermore, HIV screening of pregnant women, which has resulted in significantly lower vertical transmission of HIV in countries with high uptake, will miss a large number of women who acquired HIV after pregnancy, who have never been pregnant or are in their perimenopause or menopause. Therefore, improved HIV testing efforts for women should be addressed in future public health strategies and could include cervical cancer screening programmes, contraception services programmes, but also menopause clinics or routine healthcare attendances where blood is being drawn for other reasons. All enhanced strategies to make HIV testing more widely available including self-testing and home testing for HIV are welcome for women who feel stigmatised if they attend sexual health services. Undoubtedly, contact tracing of female partners of HIV-positive men is another strategy which could be implemented.

Research has shown that HIV is more easily transmitted sexually from men to women [3]. In a study by Thomson et al., HIV acquisition was significantly higher during pregnancy and in the post-partum period compared to women who were not pregnant: 3.75% in early pregnancy, 7.02% in later pregnancy and 4.68% in the post-partum period vs 1.25% (per year) [4] suggesting hormonal changes during pregnancy and lactation may increase women’s susceptibility to HIV. The mechanisms may be similar to the higher risk of HIV infection and transmission seen in women who use hormonal contraceptives in some, but not all, studies.

Pre-exposure prophylaxis (PrEP) has the potential to reduce HIV infection in those at risk, including women. When taken consistently, PrEP has been shown to reduce the risk of HIV infection by up to 92%. PrEP could be a particularly promising tool for women to prevent HIV acquisition as women can control its use. However, many questions remain in terms of identifying women eligible to take PrEP, mechanisms of drug delivery, acceptable and safe drug formulations during pregnancy and breastfeeding and service structures which would best answer women’s PrEP needs.

The Women Against Viruses in Europe (WAVE) promotes the welfare of HIV-positive women in Europe. The initiative involves healthcare professionals and community representatives within the European Aids Clinical Society [5]. In 2019, WAVE conducted an online survey on PrEP for women, and representatives from 38 countries completed the survey. Results showed PrEP was accessible in 17 countries for all population groups, while in a further 17 countries it was only available to men who have sex with men (MSM) and transgender persons. Almost two-thirds of country representatives confirmed the availability of a national guideline for PrEP, and five countries had specific recommendations for PrEP in women. Some of the main obstacles to overall PrEP access were lack of information, lack of political support and its cost. There were also specific obstacles for PrEP access for women, such as guidelines prioritising men who have sex with men (MSM), women not seen as a target population for PrEP, and lack of knowledge about which subgroup of women would benefit most from PrEP. Only five countries had efforts to encourage women’s access to PrEP, most of which were individually-based or initiated by local non-governmental organisations.

The WAVE survey results demonstrate that women’s access to PrEP in Europe remains limited, and there is a general lack of information about the use of PrEP both among women and among healthcare professionals. However, several of the respondents described initiatives in their countries focusing on expanding access of PrEP, including for women. Goals for the future therefore should include making PrEP available and known by women as an effective strategy to reduce HIV infection. This includes improving knowledge of healthcare providers who counsel women and women who may be at risk of HIV acquisition. PrEP strategies for women also require support by public health authorities to overcome barriers such as cost, self-esteem, knowledge and stigma, which remain considerable for women. Finally, closing the PrEP awareness gap among women is an important step in making PrEP use in women an important tool to fight new HIV infections.
